# Detection and Quantification of Neurotrophin-3 (NT-3) and Nerve Growth Factor (NGF) Levels in Early Second Trimester Amniotic Fluid: Investigation into a Possible Correlation with Abnormal Fetal Growth Velocity Patterns

**DOI:** 10.3390/jcm12124131

**Published:** 2023-06-19

**Authors:** Nikolaos Machairiotis, Dionysios Vrachnis, Nikolaos Antonakopoulos, Nikolaos Loukas, Alexandros Fotiou, Vasilios Pergialiotis, Sofoklis Stavros, Aimilia Mantzou, Georgios Maroudias, Christos Iavazzo, Christina Kanaka-Gantenbein, Petros Drakakis, Theodore Troupis, Konstantinos Vlasis, Nikolaos Vrachnis

**Affiliations:** 1 Third Department of Obstetrics and Gynecology, General University Hospital “Attikon”, Medical School, National and Kapodistrian University of Athens, 12462 Athens, Greece; nikolaosmachairiotis@gmail.com (N.M.); antonakopoulos2002@yahoo.gr (N.A.); alexandrosfotiou92@gmail.com (A.F.); sfstavrou@yahoo.com (S.S.); pdrakakis@hotmail.com (P.D.); 2 Department of Clinical Therapeutics, Alexandra Hospital, Medical School, National and Kapodistrian University of Athens, 10676 Athens, Greece; dionisisvrachnis@gmail.com; 3 Department of Obstetrics and Gynecology, University Hospital of Patras, Medical School, University of Patras, 26500 Patra, Greece; 4 Department of Obstetrics and Gynecology, Tzaneio General Hospital, 18536 Piraeus, Greece; nloux13@hotmail.com (N.L.); maroudi2011@yahoo.gr (G.M.); 5 First Department of Obstetrics and Gynecology, Alexandra Hospital, Medical School, National and Kapodistrian University of Athens, 10676 Athens, Greece; pergialiotis@hotmail.com; 6 First Department of Pediatrics, “Aghia Sophia” Children’s Hospital, Medical School, National and Kapodistrian University of Athens, 10676 Athens, Greece; amantzou@med.uoa.gr (A.M.); chriskan@med.uoa.gr (C.K.-G.); 7 Gynecologic Oncology Department, Metaxa Memorial Cancer Hospital, 18537 Piraeus, Greece; christosiavazzo@gmail.com (C.I.); ttroupis@med.uoa.gr (T.T.); 8 Department of Anatomy, Medical School, National and Kapodistrian University of Athens, 10676 Athens, Greece; vlasiskostas@gmail.com

**Keywords:** neurotrophins, nerve growth factor (NGF), neurotrophin-3 (NT-3), small for gestational age, large for gestational age, fetal growth restriction, amniotic fluid, second trimester of pregnancy, fetal metabolism, inflammation, metabolic syndrome

## Abstract

Background: Abnormal fetal growth is associated with adverse perinatal and long-term outcomes. The pathophysiological mechanisms underlying these conditions are still to be clarified. Nerve growth factor (NGF) and neurotrophin-3 (NT-3) are two neurotrophins that are mainly involved in the neuroprotection process, namely promotion of growth and differentiation, maintenance, and survival of neurons. During pregnancy, they have been correlated with placental development and fetal growth. In this study, we aimed to determine the early 2nd trimester amniotic fluid levels of NGF and NT-3 and to investigate their association with fetal growth. Methods: This is a prospective observational study. A total of 51 amniotic fluid samples were collected from women undergoing amniocentesis early in the second trimester and were stored at −80 °C. Pregnancies were followed up until delivery and birth weight was recorded. Based on birth weight, the amniotic fluid samples were divided into three groups: appropriate for gestational age (AGA), small for gestational age (SGA), and large for gestational age (LGA). NGF and NT-3 levels were determined by using Elisa kits. Results: NGF concentrations were similar between the studied groups; median values were 10.15 pg/mL, 10.15 pg/mL, and 9.14 pg/mL in SGA, LGA, and AGA fetuses, respectively. Regarding NT-3, a trend was observed towards increased NT-3 levels as fetal growth velocity decreased; median concentrations were 11.87 pg/mL, 15.9 pg/mL, and 23.5 pg/mL in SGA, AGA, and LGA fetuses, respectively, although the differences among the three groups were not statistically significant. Conclusions: Our findings suggest that fetal growth disturbances do not induce increased or decreased production of NGF and NT-3 in early second trimester amniotic fluid. The trend observed towards increased NT-3 levels as fetal growth velocity decreased shows that there may be a compensatory mechanism in place that operates in conjunction with the brain-sparing effect. Further associations between these two neurotrophins and fetal growth disturbances are discussed.

## 1. Introduction

Pathophysiological mechanisms leading to fetal growth disorders have yet to be elucidated despite the progress that has been achieved. An association has been found between fetuses with abnormal growth velocity patterns, i.e., large for gestational age (LGA) and small for gestational age (SGA), and a higher risk of adverse perinatal and long-term outcomes. LGA fetuses/neonates are defined as fetuses with birth weight (BW) greater than the 90th percentile for gestational age, while SGA fetuses/neonates are fetuses with an estimated fetal weight (EFW) below the 10th percentile at a particular gestational age [1]. They can be either structurally small due to maternal characteristics and ethnicity, or pathologically small, having failed to reach their genetic growth potential. The latter, whose condition is referred to as fetal growth restriction (FGR), present signs of severe malnutrition and hypoxia [2,3,4].

In cases of uteroplacental insufficiency and chronic hypoxia, growth restriction is thought to represent a compensatory mechanism of the fetus to the reduced maternal provision of oxygen and nutrients. The fetus responds to this state of deprivation with hemodynamic and metabolic changes. A redistribution of fetal blood circulation is noted in favor of organs such as the brain, the heart, and the adrenal glands, adjusting the blood supply required for their metabolic needs. These modifications that take place in order to protect brain metabolism constitute what is termed the brain-sparing effect [5,6].

Excessive or impaired growth has been linked with increased perinatal morbidity and mortality and with an elevated risk for a number of adverse outcomes at all subsequent stages of life, including complications in labor, cardiovascular diseases, diabetes mellitus, metabolic syndrome (MS), and neurodevelopmental defects [7,8]. The evidence shows that SGA neonates are more likely to develop behavioral problems and neurocognitive difficulties at school age compared to what is appropriate for gestational age neonates (AGA) because of deficits in neuronal connectivity and function [9,10].

Since the pathogenic mechanisms underlying abnormal fetal growth are still to be clarified, it is of utmost importance to investigate possible biomarkers that can help us understand the mechanisms leading to these conditions and which can potentially be utilized as prognostic markers. Neurotrophins are a group of signaling molecules that have been associated with neuroprotection. They include nerve growth factor (NGF), brain-derived neurotrophic factor (BDNF), neurotrophin-3 (NT-3), and neurotrophin-4/5 (NT-4/5). Their main role is the promotion of growth, maintenance and survival of neurons, actions achieved through regulation of apoptosis, survival, and differentiation of nerve cells in both the brain and peripheral nervous system. In addition, neurotrophins influence neuronal plasticity and synaptogenesis [11,12,13]. They can also regulate morphogenesis, proliferation, and apoptosis outside the nervous system, as they have been found in immune cells, adipocytes, and endocrine and endothelial cells; they also appear to play a role in the development of such organs as the kidney, the heart, and muscles [14,15,16].

NGF is a particularly important polypeptide hormone that stimulates the growth and the development of the sympathetic and central nervous systems. It contributes to the survival of cholinergic neurons in the central nervous system and to neuronal development and maintenance of the neural phenotype in the peripheral nervous system (PNS) [17]. Moreover, it has been found in human placenta, which is evidence suggestive of its potential role in placental development [18].

NT-3 is considered to be the ancestor of all neurotrophins. Its structure resembles that of NGF and BDNF [19,20,21]; however, it differs from them in that it shows higher levels within the central nervous system during fetal development than during adult life [20,22]. The fact that NT-3 transcript and protein are present in the physiological and pathological placenta right through pregnancy was established in 2009 [23], suggesting their potential role in placental development and fetal growth.

The aim of this prospective observational study was the detection and quantification of NGF and NT-3 in early second trimester amniotic fluid and investigation into a possible correlation of their concentration with LGA and SGA fetuses in order to illuminate underlying pathophysiological mechanisms leading to these extremes of fetal growth.

## 2. Materials and Methods

From women undergoing amniocentesis early in the 2nd trimester of gestation (15–22 weeks), based on indications such as advanced maternal age, increased nuchal translucency, previous history of birth defects, or detection of an anomaly in the ultrasound examination of the first or second trimester, amniotic fluid samples were collected. Following amniocentesis, the samples were centrifuged and the supernatants were then stored in polypropylene tubes at −80 °C. Twin pregnancies and pregnancies with fetuses with an abnormal karyotype or severe congenital malformations were excluded. All pregnant women were followed up until delivery. Birth weights were recorded at delivery and, subsequently, the embryos corresponding to respective amniotic fluid samples were divided into three groups: SGA, AGA, and LGA. A number of SGA fetuses were expected to be FGR and a number of LGA fetuses were severely macrosomic, both leading to early delivery, spontaneous or iatrogenic. A gestation-related weight computer program was used to allocate the centile of each neonate at the time of delivery [24].

Our study sample was composed of 12 SGA fetuses and 8 LGA fetuses matched for gestational age, sex, and maternal height and weight and compared with 31 AGA fetuses that composed the control group. The amniotic fluid samples were withdrawn from the deep-freezer and NGF and NT-3 were measured in order to compare their levels between normal full-term pregnancies (control group) and the groups of embryos with residual and excessive growth.

Amniotic fluid NGF levels were measured using the Human beta-NGF DuoSet ELISA Immunoassay kit (DY-256, R&D Systems, Minneapolis, MN, USA) and amniotic fluid NT-3 levels were measured using the Human Neurotrophin-3 DuoSet ELISA Immunoassay kit (DY-267, R&D Systems, Minneapolis, MN, USA), according to the manufacturer’s instructions. The given assays range of detection was 31.2–2000 pg/mL for both kits; however, lower concentrations were detected by implementing calibration curve extensions through zero. The sensitivity of lower calibrators was evaluated and the results were satisfactorily accurate.

Statistic evaluation of the results was carried out using the SPSS statistical package employing parametric and nonparametric methods, dependent on indications. The distribution of sample values was evaluated by regression analysis (Kolmogorov-Smirnov test). The Kruskal-Wallis test was used for comparison of NGF and NT-3 concentrations between the three groups. Median and interquartile range for quantitative variables are displayed in the results. In accordance with our study design, the confounding factors include maternal age, body mass index, duration of pregnancy, fetal sex, and multiparity. We set the level of significance at a *p* value of less than 0.05. Lastly, our analysis included a Spearman’s rank correlation coefficient between NT-3 and NGF concentrations and all other arithmetic parameters.

The Ethical Committee for Research of Attikon General University Hospital, Athens, Greece, approved the study and informed consent was obtained from all women participating in the study.

## 3. Results

Fifty-one amniotic fluid samples were measured in total. Maternal and fetal descriptive characteristics are depicted in Table 1. No statistically significant differences were observed among the three groups regarding maternal age, maternal weight, maternal height, maternal BMI, parity, mode of delivery, and neonatal sex. However, gestational age at birth and birth weight and centile, as expected, were statistically different between groups.

**Figure 1:**
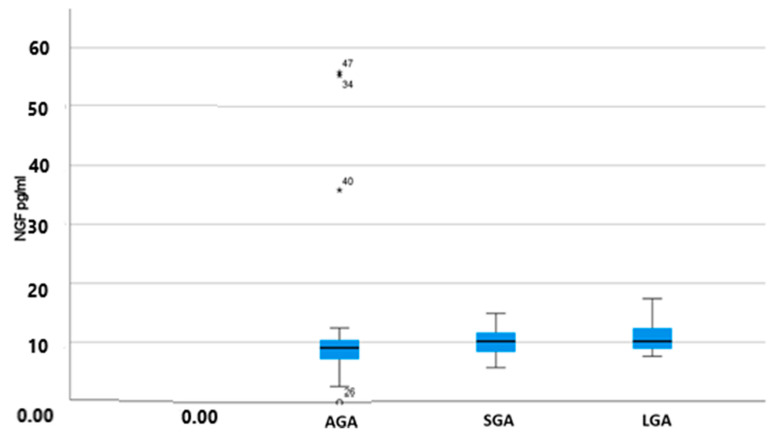
NGF median values (pg/mL) and IQR (Q1–Q3): comparison among groups (AGA, SGA, LGA). Asterisks and circle indicate outliers for the specific patients.

Table 2 and Figure 1 summarize the NGF assay results, showing the median values of NGF in the three studied groups, SGA, LGA, and AGA/Control group. Medians were used as there was no normal distribution of values. Differences were not statistically significant among the three groups (median values of 10.15 pg/mL and 10.15 pg/mL vs. 9.14 pg/mL for SGA and LGA fetuses vs. AGA fetuses, respectively; *p* = 0.254).

**Figure 2:**
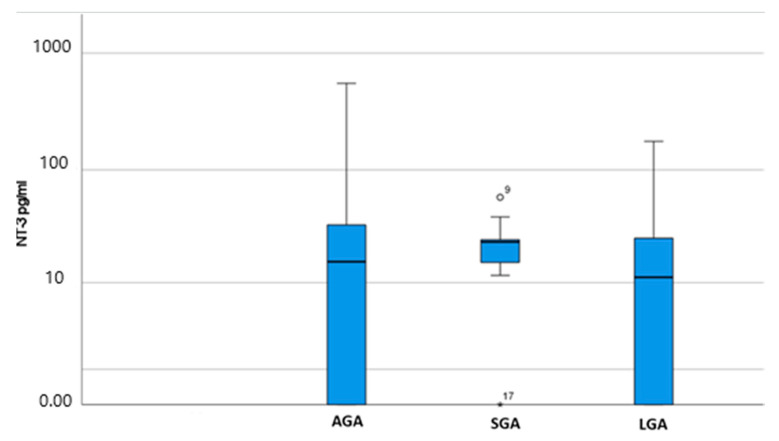
NT-3 median values (pg/mL) and IQR (Q1–Q3): comparison among groups (AGA, SGA, LGA). Asterisk and circle indicate outliers for the specific patients.

Table 3 and Figure 2 summarize the NT-3 assay results, showing the median values of NT-3 in the three studied groups, SGA, LGA, and AGA/Control group. Medians were used as there was no normal distribution of values. Differences were not statistically significant among the three groups (median values of 23.5 pg/mL and 11.87 pg/mL vs. 15.9 pg/mL for SGA and LGA fetuses vs. AGA fetuses, respectively; *p* = 0.398).

Table 4 presents the Spearman’s rank correlation coefficient between NT-3 and NGF concentrations and all other arithmetic parameters. NT-3 and NGF concentrations were not found to be statistically correlated with either the birth weight or the percentile of weight at birth. Moreover, NT-3 and NGF were not correlated to each other.

## 4. Discussion

Fetal growth disturbances remain a significant cause of adverse perinatal and long-term outcomes during all stages of life. The pathophysiology behind these conditions is yet to be fully clarified despite the progress achieved in clinical fetal medicine. To our knowledge, no human research has been conducted concerning potential NGF and NT-3 associations with fetal growth nor regarding fetal adaptation to a hostile intrauterine environment in SGA and LGA pregnancies, while data are lacking as to the possible involvement of neurotrophic factors in macrosomia. Our study aimed to confirm the presence of both neurotrophins, NGF, and NT-3 in early second trimester amniotic fluid specimens and to explore possible associations between the neurotrophin levels of this early fetal period with fetal growth disturbances confirmed at the time of birth.

Amniotic fluid is composed of fetal urine, lung excretion, and factors that can potentially influence fetal growth, thus providing a dynamic environment [25,26,27,28]. Neurotrophins have the ability to cross the blood-brain barrier and can be detected in the peripheral blood [29]. As amniotic fluid from pregnancies early in the second trimester reflects fetal serum [30,31], this composes a reliable sample to measure the concentration of these neuroproteins in the fetus and determine what associations, if any, they have with conditions in which they may be involved as evidence suggests that circulating amniotic fluid neurotrophins can influence fetal neurodevelopment during pregnancy [32].

Neurotrophins, which act as paracrine and autocrine growth factors, play a significant role in regulating morphogenesis and the development of a number of tissues. Furthermore, they mainly promote the growth and differentiation of neurons and their regulatory actions are important since early development [33,34,35]. Neurotrophins additionally control not only the generation, survival, differentiation, and death of neurons within the peripheral and central nervous system at the embryonic and postnatal stages but also neuronal maintenance later in life [36,37]. Their presence in human amniotic fluid offers the opportunity to elucidate their regulation in the fetal development throughout pregnancy [38].

In pregnancy, maternal stress increases the cortisol levels in maternal plasma, which are strongly associated with cortisol levels in the amniotic fluid [39]. Furthermore, maternal obesity increases the level of inflammatory cytokines, such as TNF-α and interleukin-8, in the amniotic fluid [40]. The above two factors strongly point to their potential to influence neuronic development through neurotropic cascade signaling, since they possess the ability to transactivate tyrosine receptor kinase receptors (TRKs) [41]. Neurotrophin effects are mediated through activation of TRKs, which subsequently triggers the mitogen-activated protein (MAP) kinase pathway, the function of this pathway being to regulate neuronal proliferation, differentiation, and apoptosis as well as neurotransmitters, neurotrophins, and hormones [42].

NGF, a polypeptide hormone, plays a critical role in the development of the sympathetic and central nervous systems. The NGF gene is localized on chromosome 1. Over three decades ago, in 1989, amniotic fluid NFG activity was found to range from below 10 pM to nanomolar, translating into clinically obtainable levels for their detection and for determination of their possible correlation with disorders involving alterations in NGF levels or activity. NGF expression in human trophoblast, deciduas, and fetal membranes has also been confirmed during the first and third trimester of pregnancy, the latter pointing to a role of this neurotrophic factor in the regulation of fetal growth and placental development [18].

Concerning NGF concentrations, our findings were similar among the studied groups, which led us to the conclusion that NGF levels are not affected by fetal nutritional status in the early second trimester, nor do they affect gestational weight at term. Of great benefit would be further studies exploring the role of the amniotic fluid NGF in fetal growth at a later gestational stage than in the early second trimester as we did in our study.

Numerous studies in the literature propose that obesity is linked to elevated NGF levels and show that overweight, obese, and morbidly obese women, as well as women with MS, tend to exhibit higher NGF levels [43]. Fetal macrosomia approximates fetal obesity. Elevated NGF levels have been linked to increased hepatic triglyceride secretion, possibly due to up-regulation of hepatic lipoprotein secretion. This indicates that NGF is implicated in pathways related to energy metabolism and body weight [43]. Thus, in obesity, high NGF levels result in elevated blood lipid concentrations and increased lipogenesis. It thus appears that NGF acts as a response protein related to alterations in adipokines and inflammation that typically characterize obesity [44]. This hypothesis is further supported by the association between NGF and adipokines (e.g., TNF-α, adiponectin, leptin), pointing to its involvement in inflammation and lipid metabolism regulation. It is conceivable that in such circumstances deviant regulation can lead to fetal macrosomia [43].

There is evidence in the literature that NGF is also related to diabetes, a clinical condition that is also characterized by inflammatory processes playing an important role in glucose tolerance and insulin sensitivity dysregulation [45]. Type 1 diabetes mellitus patients who have no complications exhibit higher serum NGF levels when compared to non-diabetics [46]. In type 2 diabetes mellitus, NGF levels are also elevated, as shown by the notably higher urinary NGF levels in these cases [47,48]. Interestingly, when comparing type 1 with type 2 diabetic patients with similar durations of hyperglycemia and HbA1c levels, type 1 patients tend to have higher NGF levels than those with type 2 diabetes [46]. Experimental research has indicated a role for NGF in the hormonal control of glucose metabolism, elevated glucose levels being observed to stimulate the release of NGF and its signaling by TrkA receptors, expressed by pancreatic β-cells; the latter promoting actin rearrangement, granule mobilization, and insulin secretion [49]. NGF also counteracts molecular alterations, while administration of NGF reduces ER stress and apoptosis in Schwann cells in diabetic peripheral neuropathy, also effectively lessening neuronal death induced by glucose fluctuation in diabetic encephalopathy [50,51,52].

Overall, diabetes is associated with elevated NGF levels. As fetal macrosomia is usually the result of fetal hyperglycemia due to maternal gestational diabetes, it was important to measure NGF levels in amniotic fluid in our study so as to draw conclusions concerning fetal NGF levels. The fact that we did not identify any statistical difference may be due to the small number of LGA cases, many of which are not a consequence of gestational diabetes. Furthermore, gestational diabetes does not become apparent until the late second trimester of pregnancy when the routine glucose tolerance test is performed. Thus, our measurements in the early second trimester may have missed NGF-level changes which emerge later on.

Evidence suggests that MS originates in the intrauterine environment. Maternal nutritional, hormonal, and metabolic status affects the fetal environment, leading to a response by the fetus that promotes survival under specific conditions. These actions that enable adaptation of the fetus to the deprived intrauterine environment can potentially predispose to the development of MS in later stages of life if the adult life environment is nutritionally rich [53]. In MS, hypertrophic adipose tissue accelerates the release of free fatty acids. This leads to peripheral insulin resistance and impairment of pancreatic β-cell insulin secretion, while the adipocytes elevate adipokine secretion, NGF, interleukin 6 (IL-6), IL-1, tumor necrosis factor-α (TNF-α), and monocyte chemoattractant protein-1 (MCP-1). MS entails development of chronic dysfunction of the hypothalamic-pituitary-adrenal (HPA) axis and decreased NGF levels. Thus, pathophysiological links between NGF levels and development of MS may begin during fetal life, particularly since it is thought that NGF regulates certain pathways possibly leading to MS development. However, there is at present a lack of integrative data on the manifold roles of this neurotrophin within tissues involved in the immunoendocrine axis as well as on whether disruption of these might induce development of MS [54]. In mammals, pancreatic β-cells are the only cells that synthesize and secrete NGF and insulin. Notably, the latter two are secreted in response to elevated glucose plasma levels, possibly pointing to an autocrine feedback loop in insulin secretion via NGF. According to the concept concerning “the fetal origins of MS”, these actions may be programmed from such an early stage as intrauterine life [44,55].

There is also conflicting evidence concerning NGF levels in amniotic fluid during pregnancy. According to the study of Xia et al., the NGF level in amniotic fluid declines as the pregnancy progresses [56]; in contrast, the study of Marx et al. showed that NGF increases with gestational age [38]. The above inconsistency of data may imply evidence of additional factors influencing NGF production. In any case, our findings do not support a possible relationship between NGF levels in the second trimester of pregnancy and gestational age at birth.

As mentioned above, the association between NGF and TNF-α suggests its implication in the regulation of inflammation. Severe SGA fetuses are by definition growth restricted and exhibit hypoxemic changes and inflammatory responses. However, since these are usually apparent in the third trimester, they are presumably not reflected in early second trimester amniotic fluid sampling. Concerning FGR cases, there is a proven strong association between FGR and adult obesity. If fat deposition is reduced in response to a suboptimal in utero environment, this is possibly mediated by factors such as NGF, and it is hypothesized to lead to an increased risk of obesity in adult life [57].

It is also of interest that increased levels of NGF have a crucial role in post-infarction cardiac remodeling [58]. NGF is critical for the survival and development of sympathetic and sensory neurons while also contributing to vasculogenesis. Characteristics of acute myocardial infarction and heart failure are alterations in the expression and activity of neurotrophic factors and their receptors. This may impair the innervation of the heart muscle while also affecting cardiomyocytes as well as endothelial and smooth muscle vascular cells.

The placenta is a unique highly vascular organ. Its hypoperfusion, which is the main mechanism for FGR, starts as early as the first trimester. In this setting, it is possible that NGF could serve as a protective factor in the event of placental hypoperfusion. The fetal myocardium is rarely affected as labor is in most cases induced before severe fetal deterioration can occur, such as reversed a-wave in ductus venosus flow or severe decelerations in cardiotocography. However, it is difficult to collect the number of cases required to evaluate this hypothesis accurately, while on the other hand, in the early second trimester such hypoxic mechanisms may not have taken place.

NT-3, the ancestor of all neurotrophins as discussed above, known to play an important role in antenatal and postnatal neuronal development, is likely to be implicated in the development and maturation of sympathetic neurons [59]. Within migratory neural crest cells, NT-3’s mitotic effect suggests that it is involved in gangliogenesis, while it exhibits autocrine and/or paracrine actions needed for the proliferation, differentiation, and survival of sympathetic neuroblasts [47]. The presence of the NT-3 transcript and protein in the physiological and pathological placenta throughout pregnancy has been confirmed in a study which investigated the presence of this molecule in placental specimens of normal pregnancies and pregnancies complicated by preeclampsia and chorioamnionitis [54]. In our small cohort study, there were only two cases complicated by gestational hypertension, one case from the AGA group who also developed gestational diabetes and another case from the LGA group. The small number of these events is not indicative and does not allow us to draw conclusions. Larger studies should be conducted in order to evaluate these results.

However, NT-3 concentrations differed between the studied groups and a clear trend was observed towards increased NT-3 levels as fetal growth velocity decreased, although without a statistical level of significance being reached. In particular, the median NT-3 concentration of LGA fetuses was lower than that of AGA fetuses (11.87 pg/mL vs. 15.9 pg/mL, respectively), whereas the median NT-3 concentration of SGA fetuses was higher than that of AGA fetuses (23.5 pg/mL vs. 15.9 pg/mL, respectively), leading to approximately double levels in SGA fetuses compared to LGA ones.

Published data show that NT-3 and BDNF can protect neurons against metabolic and excitotoxic insults, which lead to excessive activation of neuronal aminoacid receptors. Thus, both may serve in calcium channel stabilization and neuroprotective functions in the brain [60]. It can hence be assumed that these neurotrophins are induced in the event of severe growth restriction as a compensatory mechanism along with the brain-sparing effect.

In contrast to BDNF and NGF, there is a paucity of experimental research on how NT-3 may be involved in energy homeostasis regulation. NT-3 may, in fact, modulate mitochondrial dynamics, causing an increase in mitochondrial density along the embryonic sensory axons [61]. One study investigating the pathology of diabetes has examined the effects of NT-3 on cell energy metabolism, while another showed that NT-3 counteracted the depolarization of the mitochondrial inner membrane in sensory neurons of streptozotocin-treated mice and that it was able to neutralize the elevation of blood glucose levels observed in obese diabetic mice [62,63].

Our previous research has shown that severely impaired fetal growth induces increased fetal BDNF production, an adaptive mechanism reacting to a hostile intrauterine environment, thus accelerating fetal brain development and maturation [13]. Although in the present study we aimed to assess the two other main neurotrophins, NGF and NT-3, and to investigate whether there is a similar role in fetal growth disturbances, our results do not support this hypothesis regarding NGF as the concentrations of the three studied groups were similar. Concerning NT-3, the comparison between the three studied groups showed a trend towards increased levels as fetal growth velocity decreased, but it failed to reach statistical significance.

FGR may be attributed to anatomical or functional diseases occurring in the fetal-placental unit, causing an adaptation of the fetal circulation to redistribute fetal blood flow, nutrient supply, and oxygen to the vital organs. Because this brain-sparing effect may take place in full-term FGR infants, late non-severe FGR infants and AGA infants are likely to display similar circulating neurotrophin levels. This may account for the non-significant differences in the studied neurotrophins among the groups. Nevertheless, there is still uncertainty regarding the time of initial triggering in early or very severe FGR fetuses and macrosomic fetuses, a period that could well be much later than the early second trimester that we studied.

To our knowledge this is the first study that detected both NGF and NT-3 in early second trimester amniotic fluid and investigated their association with excessive and impaired fetal growth. Amniotic fluid, as explained above, composes the most reliable specimen to measure the concentrations of neurotrophins during this specific intrauterine period. Moreover, another strength of our study was its prospective design, which limits selection bias, rendering the results accurate and representative of our population.

Several limitations apply to our study and may have masked the role of these neurotrophins. The main limitation was the number of cases included due to the prospective nature of the study along with the present-day infrequency of amniocenteses for prenatal diagnosis. Thus, the increased use of non-invasive prenatal testing made the accumulation of a sufficient number of amniotic fluid cases difficult. For the same reason we were unable to conduct a subgroup analysis with cases of SGR and/or macrosomia, where the results might have been different.

## 5. Conclusions

To the best of our knowledge, this study is the first to confirm the presence of NGF and NT-3 in the amniotic fluid of early second trimester human pregnancies and to simultaneously examine their correlation with different fetal growth velocity patterns. Previous studies have shown that impaired growth can potentially lead to increased production of other neurotrophins. According to our findings, no evidence of such a correlation exists for NGF and NT-3, only a non-statistically significant difference in concentrations having been observed among the studied groups. This is possibly attributable to the activation of mechanisms protecting the vital organs during such extreme conditions (brain-sparing effect), which preserve brain metabolism until a decompensation might occur. Nevertheless, regarding NT-3, a trend was observed towards increased NT-3 levels as fetal growth velocity decreased; this may be a compensatory mechanism in place along with the brain-sparing effect. The prominent NT-3 levels in SGA fetuses were almost double compared to LGA (23.5 pg/mL to 11.87 pg/mL, respectively).

Further larger studies are required to validate our results or reveal an association that could not be indicated in our study, so as to provide greater insight into the association between these two neurotrophins and fetal growth disturbances. Understanding the mechanisms underlying excessive and impaired growth will allow us to identify these pathologies at earlier stages, thus providing better monitoring, diagnosis and timely interventions.

## Figures and Tables

**Table 1:** Comparative characteristics between groups (SGA, LGA and AGA) presented as median values (Q1–Q3) or frequencies.

	AGA (*n* = 31)	SGA (*n* = 12)	LGA (*n* = 8)	*p*-Value
Maternal age (years)	37 (18–43)	35 (29–43)	37.5 (31–41)	0.295
Maternal weight (kg)	59 (47–92)	63 (49–105)	62 (58–100)	0.223
Maternal height (cm)	161 (155–175)	166 (152–174)	170 (162–174)	0.088
Maternal BMI	21 (20–25)	22 (20–27)	22 (21–26)	0.621
Parity (nulliparity)	16/28	7/11	6/7	0.394
Gestational age (days)	275 (261–285)	273 (240–277)	267 (261–274)	0.019
Neonatal birth weight (g)	3265 (2860–3750)	2640 (1750–2860)	3775 (3550–3950)	<0.001
Neonatal sex (female)	5/30	2/12	1/8	>0.999
Mode of delivery	19/28	7/12	2/8	0.111
Percentile	45 (20–74)	3.5 (1–9)	93 (92–96)	<0.001

**Table 2:** Amniotic fluid NGF mean values in the three studied groups: SGA, LGA and Control.

Group	N	NGF Median Value (Q1–Q3)	*p*-Value
SGA	12	10.15 (8.35–11.65)	0.254
LGA	8	10.15 (8.87–12.4)	
AGA (Control)	31	9.14 (7.12–10.8)	

**Table 3:** Amniotic fluid NT-3 mean values in the three studied groups: SGA, LGA and Control.

Group	N	NT-3 Median Value (Q1–Q3)	*p*-Value
SGA	12	23.5 (15.5–24.6)	0.398
LGA	8	11.87 (0–25.85)	
AGA (Control)	31	15.9 (0–35.2)	

**Table 4:** Spearman’s rank correlation coefficient between NT-3 and NGF concentrations and all other arithmetic parameters. * Correlation is significant at a level < 0.05.

	NT-3	NGF	Age	Weight	Height	Gestational Age	Birth Weight	Percentile
NT-3	1	0.317	0.159	–0.109	0.122	–0.104	–0.218	–0.043
NGF		1	0.047	0.130	0.066	–0.157	0.265	0.111
Age			1	0.127	–0.092	0.131	0.009	–0.212
Weight				1	0.447 *	0.164	0.109	0.350
Height					1	0.118	0.465	0.110
Gestational age						1	0.487 *	0.006
Birth weight							1	0.528 *
Percentile								1

## Data Availability

Data available on request due to restrictions eg privacy or ethical.
